# Task-evoked pupillometry provides a window into the development of short-term memory capacity

**DOI:** 10.3389/fpsyg.2014.00218

**Published:** 2014-03-13

**Authors:** Elizabeth L. Johnson, Alison T. Miller Singley, Andrew D. Peckham, Sheri L. Johnson, Silvia A. Bunge

**Affiliations:** ^1^Department of Psychology, University of California BerkeleyBerkeley, CA, USA; ^2^Helen Wills Neuroscience Institute, University of California BerkeleyBerkeley, CA, USA

**Keywords:** short-term memory, digit span, task-evoked pupillary response, pupillometry, development

## Abstract

The capacity to keep multiple items in short-term memory (STM) improves over childhood and provides the foundation for the development of multiple cognitive abilities. The goal of this study was to measure the extent to which age differences in STM capacity are related to differences in task engagement during encoding. Children (*n* = 69, mean age = 10.6 years) and adults (*n* = 54, mean age = 27.5 years) performed two STM tasks: the forward digit span test from the Wechsler Intelligence Scale for Children (WISC) and a novel eyetracking digit span task designed to overload STM capacity. Building on prior research showing that task-evoked pupil dilation can be used as a real-time index of task engagement, we measured changes in pupil dilation while participants encoded long sequences of digits for subsequent recall. As expected, adults outperformed children on both STM tasks. We found similar patterns of pupil dilation while children and adults listened to the first six digits on our STM overload task, after which the adults' pupils continued to dilate and the children's began to constrict, suggesting that the children had reached their cognitive limits and that they had begun to disengage from the task. Indeed, the point at which pupil dilation peaked at encoding was a significant predictor of WISC forward span, and this relationship held even after partialing out recall performance on the STM overload task. These findings indicate that sustained task engagement at encoding is an important component of the development of STM.

## Introduction

The ability to maintain information for a short period of time, known variably as short-term memory (STM) or the storage component of working memory, increases over childhood (for meta-analysis see Simmering and Perone, 2013). STM capacity is tied to the ability to perform complex cognitive tasks, such as reading and math (Baddeley, 1992; Cowan et al., 2011), and the development of STM capacity partially governs age-related gains in higher-order cognitive functions (Bayliss et al., 2005; Magimairaj and Montgomery, 2012). The goal of the present study was to gain mechanistic insights into developmental changes and individual differences in STM capacity.

One of the most commonly used indices of STM in children is the digit span task, a measure of verbal STM (Bayliss et al., 2005; Cowan et al., 2005). The digit span task requires the encoding and immediate serial recall of a list of numbers presented aurally, and the length of an individual's span depends on how well s/he can attend to, rehearse, and subsequently repeat back the stimuli. The ability to remember long lists in simple span tasks has been validated as a robust correlate of higher-order cognitive functions as measured by complex span tasks in children (Cowan et al., 2005) and adults (Unsworth and Engle, 2007a,b). Age-related changes and individual differences in digit span could in theory reflect differences in cognitive resource allocation at encoding, rehearsal, and/or recall. Here, we sought to assess the extent to which age-related changes and individual differences in STM capacity could be explained by differences in cognitive effort during stimulus encoding, as measured via the task-evoked pupillary response to cognitive load (Hess and Polt, 1964; Beatty, 1982; Beatty and Lucero-Wagoner, 2000; Karatekin, 2007; Laeng et al., 2012).

Pupil size is governed both by ambient light levels and physiological arousal (Kahneman, 1973; Beatty, 1982; Beatty and Lucero-Wagoner, 2000; Karatekin, 2007; Laeng et al., 2012). Pupil dilation related to physiological arousal is mediated by the simultaneous activation of sympathetic pathways and inhibition of parasympathetic pathways (Beatty and Lucero-Wagoner, 2000), and evidence suggests that task-evoked pupil dilation results from cortical inhibition of the parasympathetic oculomotor nucleus (Wilhelm et al., 1999; Steinhauer et al., 2004). During a state of heightened attention, neurons in the locus coeruleus fire rapidly, supplying high levels of noradrenaline to numerous targets throughout the body, including both the eyes and brain. In the eye, this neurotransmitter mediates pupil dilation; in the brain, it regulates attention through its modulatory effects on brain activity (see Gilzenrat et al., 2010; Laeng et al., 2012; Donner and Nieuwenhuis, 2013; Eldar et al., 2013).

Task-evoked pupil dilation in well-controlled experimental settings has been referred to variably as a peripheral marker of heightened attention, mental effort, or allocation of cognitive control when the task prompts focus or conscious engagement. Kahneman (1973) described it as reflecting the “intensive aspect” of attention; more recently, Gilzenrat et al. (2010) have described task-evoked pupillary dilation as reflecting task engagement. Indeed, a large body of research provides compelling evidence that task-evoked pupil dilation is sensitive to cognitive load (Beatty, 1982; Beatty and Lucero-Wagoner, 2000). Beginning with Kahneman and Beatty (1966), researchers have consistently shown that adults' pupils dilate incrementally with each digit encoded in a digit span task until the length of the digit sequence exceeds STM capacity, at which point pupil size begins to plateau or diminish (Kahneman et al., 1968; Peavler, 1974; Granholm et al., 1996, 1997; Cabestrero et al., 2009). Pupils also tend to constrict during recall as items are offloaded from STM (Kahneman and Beatty, 1966; Cabestrero et al., 2009). These findings are consistent with the idea that cognitive resources are dedicated in a manner proportionate to the cognitive load.

Pupil dilation patterns have also been used to examine individual differences in cognitive functioning among adults. Ahern and Beatty (1979, 1981) showed that cognitively higher-functioning adults—as defined based on their scores on the Scholastic Aptitude Test—exhibited consistently smaller dilation amplitudes on STM, mental multiplication, and sentence comprehension tasks than lower-functioning adults. These patterns of pupil dilation were interpreted as indices of mental effort, suggesting that performance of the same cognitive task was less challenging for higher-functioning adults. Taken together, the results of prior studies validate pupil dilation as a measure of task engagement, with pupils dilating as cognitive effort is expended.

Simmering and Perone (2013) have argued that the field of cognitive development would benefit from research linking theory to real-time behavior; specifically, they call for approaches that combine evidence from “micro-behavior”—i.e., indices of mechanisms underlying cognitive processes—and “macro” measures such as performance accuracy. We propose that task-evoked pupillometry represents a “micro” index of mental effort that can be used to probe developmental changes in task engagement. Given its high temporal resolution, well-validated use in studies of adult cognition, and non-invasive nature, task-evoked pupillometry has the potential to provide important insights with regard to cognitive development (cf. Karatekin, 2007; Laeng et al., 2012).

Thus far, there have been only a few studies of task-evoked pupillometry involving children (Boersma et al., 1970; Karatekin, 2004, 2007; Karatekin et al., 2007a,b; Chatham et al., 2009), and only one of these studies involved a digit span task (Karatekin, 2004). In this study, 10-year-olds (*n* = 15) and young adults (*n* = 21) performed a digit span task in which they listened to sequences of 4, 6, and 8 digits. Although the 10-year-olds did not perform as well as the adults on either the 6- or 8-digit sequences, their patterns of pupil dilation differed only when they encoded the 8-digit sequences (Karatekin, 2004). On these long sequences, children exhibited shallower mean rates of dilation per digit than did adults, which the authors interpreted as indicating that they allocated fewer cognitive resources to the task.

Here, we sought to more closely examine the relationships between task engagement at encoding and developmental changes and individual differences in STM capacity. To this end, we measured pupil diameter continuously as participants encoded digit sequences that exceeded typical STM capacity, i.e., an STM overload task. If, as the results of Karatekin (2004) suggest, children are unable to recruit cognitive resources sufficient to encode at high loads, then their pupils should stop dilating (Cabestrero et al., 2009) and/or constrict (Peavler, 1974; Granholm et al., 1996) earlier in the sequence as compared to adults. Seeking to explore the relationship between these task-evoked pupillary responses and differences in STM capacity, we also administered the forward span task from the Digit Span subtest of the Wechsler Intelligence Scale for Children (Wechsler, 2003) to both children and adults. We hypothesized that if the point at which pupil diameter asymptotes is related to the amount of information encoded into STM, then this value should be related to STM capacity.

## Methods

### Participants

Sixty-nine healthy children (36 males, 33 females; ages 7.5–14.0 years, mean 10.6 ± 1.1 years) and 54 healthy adults (27 males, 27 females; ages 18.3–60.8 years, mean 27.5 ± 10.8 years) participated in this study.^1^ Children were recruited through the Berkeley Chess School outreach program at public schools in Oakland, CA, or surrounding San Francisco Bay Area communities, and thanked via a classroom gift by request of the school administration. Adults were recruited from the University of California, Berkeley, or the San Francisco Bay Area via advertisements, and received monetary compensation or—for UC Berkeley students in the Research Participation Pool—course credit. All participants had normal or corrected-to-normal vision and hearing, and were fluent in English.

### Behavioral forward digit span

To assess STM capacity, we used the forward span task in the Digit Span subtest on the Wechsler Intelligence Scale for Children—Fourth Edition (WISC-IV; Wechsler, 2003). The forward span task is a commonly used behavioral measure of verbal STM in multiple populations (Kane et al., 2004; Bayliss et al., 2005; Cowan et al., 2005; Alloway et al., 2009). The Digit Span subtest procedure is identical in the children and adult Wechsler test batteries; we chose to use the WISC subtest across age groups to keep the digit lists constant. Participants are read a series of digits (e.g., “9, 4, 2”) at a rate of one digit per second and are asked to repeat the digits back to the experimenter in the same serial order presented. Two trials are presented at each span length, starting with two digits per trial. If the participant repeats at least one of the two trials of the same sequence length successfully, the experimenter presents two trials of a sequence that is one digit longer. This procedure continues until the participant misses both trials of a particular span length or completes the trials with the maximum 9-digit span.

In tests of verbal STM, healthy adults remember an average of seven digits, plus or minus two (Miller, 1956); children tend to remember fewer digits than adults (Simmering and Perone, 2013). An individual's STM span is calculated as the length of the longest sequence of digits successfully repeated back to the experimenter, for a maximum of 9. The forward total score reflects the number of trials each participant completed correctly, for a maximum of 16.

### STM overload task

Following administration of the WISC forward digit span, participants completed a computerized STM overload task while undergoing eyetracking. Our task was adapted from Peavler (1974), Granholm et al. (1996, 1997), Karatekin (2004), and Cabestrero et al. (2009). As in the WISC task, participants heard a sequence of digits, presented at the rate of one digit per second, and were asked to repeat them back immediately in the same order presented (Wechsler, 2003). In our adaptation of the task, participants completed a total of four trials, all involving the same number of digits. Children were asked to encode sequences of nine digits, whereas adults were asked to encode sequences of 11 digits (the same nine digits as for the children, with two additional digits added at the end of the sequence). These digit sequence lengths were chosen because they exceed average WISC forward spans, allowing us to examine pupillary responses once participants surpassed their individual encoding limitations (Granholm et al., 1996, 1997; Karatekin, 2004; Cabestrero et al., 2009). For the present purposes, we were interested in average pupil dilation and subsequent serial recall accuracy for each digit.

All participants were informed that they would hear a series of numbers. They were instructed to remember the digits as presented and then do their best to recall the full sequence of digits in the correct order. Each trial began with a 1-s auditory cue (“memorize”), alerting participants to the beginning of a trial. After the last digit for the trial was presented, the word “recall” signaled the participant to repeat the numbers back; as in the WISC forward digit span, the recall phase was self-paced. Participants completed all four trials irrespective of recall accuracy. The experimenter manually recorded participants' responses during the recall phase.

Both children and adults completed the same two practice trials before the experimental trials: a 3-digit trial followed by a 5-digit trial. They were permitted to repeat this round by request. After practice, participants underwent a 5-point eyetracking calibration procedure, and then began the experimental trials. Within each age group, all participants completed the same four experimental trials, with the order of trials randomized.

Participants were instructed to look at a 1 × 1 inch fixation cross in the middle of the screen, presented in white on a black background, throughout the computer task. This design permitted the recording of pupil data at fixed luminance for the duration of the task, ensuring that pupillary responses were independent of pupillary light reflexes (Beatty, 1982; Beatty and Lucero-Wagoner, 2000). To allow participants' pupil diameters to return to a neutral baseline before the start of each trial (e.g., Cabestrero et al., 2009; van der Meer et al., 2010), we programmed the task in such a way that it proceeded automatically to the next trial only after the eyetracker had captured 2 s of continuous data.

### Eyetracking apparatus

Stimuli were presented using the Tobii E-Prime Software Extensions (Psychology Software Tools, Pittsburgh, PA), which syncs the timing of stimulus presentation with a second computer that records pupil data. Participants were seated comfortably in front of the Tobii T120 Eye Tracker (17-inch monitor, 1280 × 1024 pixel resolution); distance was calibrated individually so that each participant focused on the middle of the screen, within a range of 50–80 cm. The Tobii T120 built-in camera captures data with a temporal resolution of 120 Hz, producing a data point every 8.3 ms, and average spatial resolution of 0.3° of visual angle. Because the camera can automatically compensate for small head movements (within a 30 × 22 cm area at 70 cm distance), participants' heads were not restrained. The camera simultaneously recorded the pupil diameter of the left and right eyes.

### Data analyses

Nineteen children and eight adults were excluded from the sample due to insufficient recording of eyetracking data, yielding data from 69 children and 54 adults. We considered recordings insufficient if pupil data were absent across all four trials of at least one digit or while hearing the “memorize” cue (i.e., the cue period), or if less than 25% of data remained overall after cleaning the data to remove artifacts (adapted from Granholm et al., 1996; Siegle et al., 2011). These were cases of either technical error or excessive blinking or head motion on the participant's part, and so using such stringent cutoffs permitted us to perform analyses without need for interpolating data points to fill gaps in data collection.

Data were cleaned using a local fit procedure. We manually inspected graphic displays of a subset of data in each group sample for artifacts (e.g., partial eyelid closures, apparent changes in diameter resulting from motion), and then implemented a computer algorithm to automate this process for all subjects. A local regression model was applied to the full datasets (loess model; Cleveland et al., 1992), such that data points were removed from analysis if they fell out of the range of five standard errors above or below the locally defined, weighted mean. We applied this process separately to the raw pupil diameter of each eye, fitting locally over 400-ms segments of data around each diameter data point.^2^ Because subjects' heads were not restrained, we also applied this procedure to the mean distance between subjects' eyes and the camera. We used a more conservative fit based on 200 ms around each distance data point in order to pick up artifacts due to abrupt changes in head position. Overall, data were discarded if they fell out of range in either eye based on pupil diameter, or based on distance; fewer than 4% of data points were removed in this procedure.

To measure pupil dilation during encoding, we calculated the average pupil diameter across both eyes at each remaining data point (8.3 ms). Data for one eye were used when data for both were not available. We then calculated the mean diameter over each second, time-locked to the presentation of each stimulus, averaged across the four experimental trials. This procedure yielded one data point for the “memorize” cue, and either nine or eleven data points for the digit sequence, depending on whether the participant was a child or an adult.

The absolute diameter of the pupil at rest is known to decrease from childhood into adulthood. This age-related change is posited to reflect a gradual decrease over childhood in the influence of the sympathetic branch concurrent with a decrease in central inhibition of the parasympathetic pathway (Karatekin et al., 2007a). Thus, to compare patterns of pupil dilation between children and adults, it is necessary to control for these differences in baseline pupil diameter.

Task-evoked pupil dilation was defined as the percentage of dilation at each digit, over 1 s, relative to the mean pupil diameter over the 1-s cue period, i.e., dilation_digit_ = (diameter_digit_-diameter_cue_) / diameter_cue_ (Karatekin, 2004; also Hess and Polt, 1964; Beatty and Lucero-Wagoner, 2000). Pupil dilation data were submitted to a mixed-model, repeated-measures analysis of variance (ANOVA), with digit as the within-subjects factor and age group as the between-subjects factor. Planned *post-hoc* comparisons between dilation at each digit and the next consecutive digit in the sequence were performed within each age group.

Recall accuracy was defined as the proportion of digits correctly recalled as a function of serial position on the STM overload task (Cowan et al., 2005). If a participant correctly recalled the first digit on all four trials, s/he was given an accuracy of 1 on the first digit. If, however, a participant correctly recalled a digit on three of the four trials, and missed it or recalled it incorrectly on one trial, s/he was given an accuracy of 0.75 for that digit. This procedure yielded values of 1, 0.75, 0.5, 0.25, or 0 for each digit. We conducted a mixed-model, repeated-measures ANOVA, and performed *post-hoc* comparisons between each digit and the next digit in the sequence within each age group. We also conducted regression analyses to further explore the relationships between measures of STM capacity and pupillary dilation at encoding, controlling for age group.

## Results

### Age-related differences in STM

**Figure 1:**
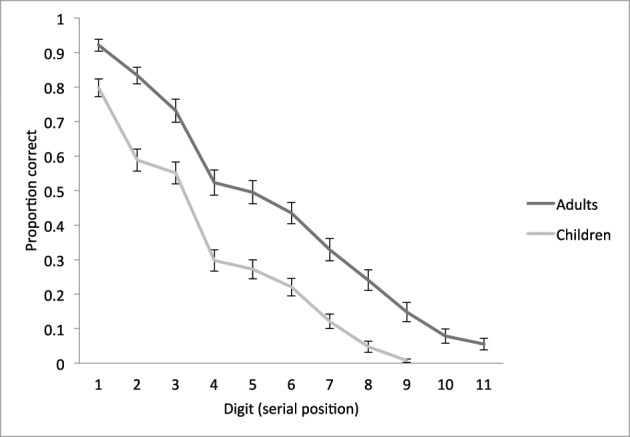
**Behavioral performance on the STM overload task**. Mean proportion of digits correctly recalled as a function of serial position, plotted separately for children and adults. Error bars represent standard mean error.

First, we tested for group differences in STM capacity on the WISC digit span test and on our computerized STM overload test. As expected, adults had significantly higher WISC forward spans and scores than children, *t*_span(115.1)_ = 7.6, *t*_score(117.8)_ = 7.9; both *p* < 0.001 (Table 1). On our STM overload task, adults recalled more digits than children (Figure 1, Table 1). A 9 (digit: 1 through 9) × 2 (age group) ANOVA revealed significant main effects of digit, *F*_(8, 960)_ = 258.92, *MSE* = 0.03, *p* < 0.001, η^2^ = 0.68, and age group, *F*_(1, 120)_ = 68.87, *MSE* = 0.15, *p* < 0.001, η^2^ = 0.37.

**Table 1:** **Descriptive statistics for WISC, pupillary, and recall accuracy data by age group**.

	**Adults**	**Children**	**Group differences**
	**Mean (*SD*)**	**Mean (*SD*)**	
**WISC FORWARD SPAN TASK**			
Span	7.19 (1.23)	5.47 (1.26)	*t*_(115.11)_ = 7.56, *p* < 0.001
Total score	11.41 (2.12)	8.24 (2.33)	*t*_(117.84)_ = 7.86, *p* < 0.001
**STM OVERLOAD TASK**			
***Encoding phase***			
Mean pupil diameter in mm			
Cue	3.81 (0.57)	3.99 (0.47)	
Digit 1	3.88 (0.60)	4.06 (0.48)	
Digit 2	3.93 (0.63)	4.08 (0.52)	
Digit 3	3.94 (0.62)	4.13 (0.51)	
Digit 4	3.98 (0.61)	4.15 (0.53)	
Digit 5	4.01 (0.62)	4.19 (0.51)	
Digit 6	4.09 (0.63)	4.21 (0.51)	
Digit 7	4.12 (0.64)	4.20 (0.52)	
Digit 8	4.14 (0.65)	4.17 (0.51)	
Digit 9	4.14 (0.66)	4.15 (0.54)	
Digit 10	4.15 (0.66)	n/a	
Digit 11	4.13 (0.67)	n/a	
Digit-at-peak dilation	7.65 (1.81)	6.10 (2.02)	*t*_(118.73)_ = 4.46, *p* < 0.001
***Recall phase***			
Total correct	4.79 (1.35)	2.90 (1.13)	
Proportion correct	0.44 (0.12)	0.32 (0.13)	*t*_(114.82)_ = 4.99, *p* < 0.001

*WISC and recall phase data were missing for one child. Digit-at-peak dilation computations are based on data from digits 1 to 9. Independent-samples t-tests were performed on variables that were standardized for comparison across age groups*.

Both groups exhibited a primacy effect, such that proportion of correctly recalled digits was high at the beginning of the digit sequence and diminished with each additional digit (i.e., serial position), consistent with prior research on immediate serial recall (Kane et al., 2004; Unsworth and Engle, 2007a,b). In adults, there were significant incremental decreases from positions 1 to 2, 2 to 3, 3 to 4, 6 to 7, 7 to 8, 8 to 9, and 9 to 10 [all *t*_(53)_ > 3.0, *p* < 0.01]; and in children, from positions 1 to 2, 3 to 4, 6 to 7, 7 to 8, and 8 to 9 [all *t*_(67)_ > 2.7, *p* < 0.01]. A follow-up one-way ANOVA showed that adults were significantly more accurate than children on all digits, all *p* < 0.001, and an independent samples *t*-test confirmed that adults recalled 12% more digits than children overall (*p* < 0.001, see Table 1). This finding is consistent with prior literature on the development of STM, showing that capacity increases with age from childhood into adulthood (Simmering and Perone, 2013).

Next, we used partial correlation analyses to test whether the standardized WISC digit span subtest and our STM overload task elicited similar behaviors, controlling for age group. This analysis showed that recall accuracy on the STM overload task was significantly, albeit modestly, correlated with WISC score after controlling for group [*r*_(119)_ = 0.19, *p* < 0.04]. The partial correlation between recall accuracy and WISC span, however, did not retain significance [*r*_(119)_ = 0.14, *p* < 0.12].

These findings suggest that the cognitive factors that contribute to performance on our STM overload task overlap partially with those of the standard digit span task, in which the length of the test sequence increases only after mastery is demonstrated at a particular sequence length. Indeed, behavioral performance on a memory test reflects the combined outcome of cognitive processes operating during encoding, maintenance, and retrieval. Given the high temporal resolution of pupillometry, by contrast, it is possible to examine measurements taken during a specific task phase. Here, we probe the relationships between STM capacity and pupil dilation during the encoding phase of our STM overload task.

### Age-related differences in pupil dilation at encoding

**Figure 2:**
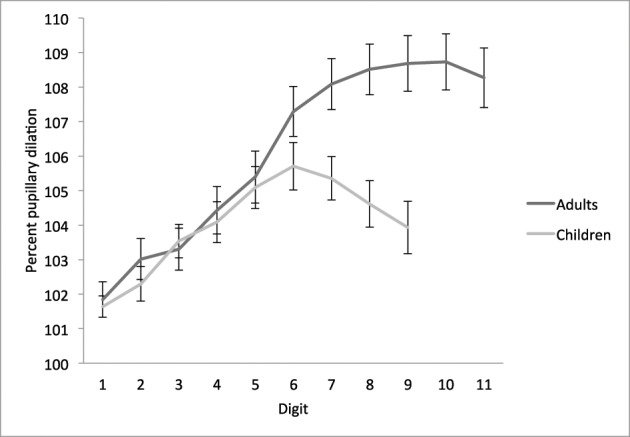
**Temporal dynamics of pupil dilation and constriction on the STM overload task**. Mean percentage of pupil dilation for each digit relative to mean pupil diameter over the cue period (set to a starting point of 100%; Karatekin, 2004), by age group. Adults encoded four sequences of 11 digits each, and children encoded four sequences of 9 digits each. Error bars represent standard mean error.

In accordance with our research aim of investigating the relationship between task-evoked pupillary responses and STM capacity, we tested for group differences in dilation relative to the cue period immediately prior to task. Consistent with prior work (Karatekin, 2004; also Beatty and Lucero-Wagoner, 2000), children had larger pupils at all timepoints than adults (Table 1); thus, we plotted pupil dilation in terms of percentage change from the cue period (Figure 2).

A 9 (digit) × 2 (age group) ANOVA revealed significant main effects of digit, *F*_(8, 968)_ = 59.24, *MSE* = 7.23, *p* < 0.001, η^2^ = 0.33, and age group, *F*_(1, 121)_ = 4.09, *MSE* = 168.03, *p* < 0.05, η^2^ = 0.03, and a significant digit × group interaction, *F*_(8, 968)_ = 13.51, *MSE* = 7.23, *p* < 0.001, η^2^ = 0.10. Both age groups demonstrated an increase in pupil dilation as a function of digit, to a point. Adults' pupils showed incremental increases from cue to digit 1, and digits 1 to 2, 3 to 4, 4 to 5, 5 to 6, and 6 to 7 [all *t*_(53)_ > 2.8, *p* < 0.01], and continued to dilate until almost digit 9 on average (8.7 ± 2.2). Children's pupils dilated until digit 6 on average (6.1 ± 2.0), with incremental increases from cue to digit 1, digit 2 to 3, and digit 4 to 5, [all *t*_(68)_ = 2.7, *p* < 0.01], and a marginally significant increase from digit 1 to 2 [*t*_(68)_ = 2.0, *p* = 0.05]. In contrast, a significant decrease was observed from digit 7 to 8, *t*_(68)_ = 2.1, *p* < 0.05.

A one-way ANOVA with age group as the between-subjects factor confirmed that adults' pupils were significantly more dilated than children's while encoding digits 7, 8, and 9 (all *p* < 0.01), indicating that where adults' pupil diameters continued to dilate or reached a stable plateau, children's pupils reached an asymptote or began to constrict. The age groups did not differ significantly in pupil dilation on digits 1 through 6 (all *p* > 0.12), suggesting a similar rate of dilation within the constraints of STM capacity.

To directly compare the latency to peak pupil dilation—i.e., digit-at-peak—between groups, we also conducted a planned comparison based on the digit (1–9) at which pupils reached maximum dilation. Adults' maximum pupil dilation occurred on average at digit 7.7 ± 1.8, which was significantly greater than children's maximum at digit 6.1 ± 2.0, *t*_(118.7)_ = 4.5, *p* < 0.001 (Table 1).

### Relationships between pupil dilation and STM

The correspondence between average digit-at-peak values (7.7 and 6.1 for adults and children, respectively) and average WISC spans (7.2 and 5.5) hints at a relationship between STM capacity and the dynamics of pupil dilation during STM encoding. To test this hypothesis directly, we first conducted linear regression analyses between the pupillary measure of digit-at-peak dilation and each behavioral STM measure: recall accuracy on the STM overload task, WISC span, and WISC score. Digit-at-peak was significantly correlated with all three measures (β_recall_ = 0.30, β_span_ = 0.38, β_score_ = 0.37; all *p* ≤ 0.001). The correlation between digit-at-peak and each WISC measure retained significance after partialing out recall accuracy on the STM overload task [*r*_span(119)_ = 0.30, *r*_score(119)_ = 0.29; both *p* = 0.001].

Next, we measured the extent to which individual variability in digit-at-peak explained individual differences in STM capacity, controlling for age group. In a multiple regression analysis, we modeled STM capacity as a function of digit-at-peak and group. This analysis revealed a strong effect of group on all three STM measures, as expected, as well as an independent contribution of digit-at-peak to each measure, *p* < 0.05 (see Table 2 for full results). These results indicate that cognitive resource allocation at encoding, as measured by the point of maximal pupil dilation on our STM overload task, can explain individual differences in STM capacity on a standard digit span task.

**Table 2:** **Multiple regression analyses for WISC score, WISC span, and recall accuracy**.

	***B***	***SE B***	**β**
**WISC FORWARD SPAN**			
Digit-at-peak	0.14	0.06	0.19^*^
Group	−1.50	0.24	−0.50^**^
**WISC FORWARD TOTAL SCORE**			
Digit-at-peak	0.24	0.10	0.18^*^
Group	−0.28	0.43	−0.51^**^
**STM OVERLOAD RECALL ACCURACY**			
Digit-at-peak	0.01	0.01	0.18^*^
Group	−0.10	0.02	−0.35^**^

* p < 0.05,

** p < 0.001

## Discussion

Consistent with decades of prior research in adults, the present results corroborate a close link between cognitive demands imposed by the digit span task and task-evoked pupil dilation (Kahneman and Beatty, 1966; Kahneman et al., 1968; Peavler, 1974; Granholm et al., 1996, 1997; Cabestrero et al., 2009), and show that children also exhibit this link (also Karatekin, 2004). Our findings extend prior work in two ways. First, we provide evidence that the children disengaged from the task as soon as the cognitive load surpassed their STM capacity, whereas adults stayed engaged while encoding additional items beyond their span. Second, we show that the point at which pupil dilation peaks is related to STM capacity—independent of age, and even after partialing out recall accuracy on the STM overload task.

With our STM span overload paradigm, we obtained similar trajectories of pupil dilation for children and adults until the sixth digit, after which the age groups diverged. Whereas adults showed dilation during encoding up to the ninth digit and then exhibited a plateau in pupil diameter until the end of the 11-digit sequence, children's pupils plateaued from digit 6 to 7, constricted from 7 to 8, and then plateaued until the end of the 9-digit sequence. In contrast to Karatekin (2004), who showed that children exhibited shallower dilation than adults during encoding of an 8-digit sequence, this finding shows children and adults dilate at similar rates up to digit 6, after which the groups' dilation patterns diverge.

Analyses focused on digit-at-peak revealed a significant relationship between the ordinal number corresponding to the digit at which maximal pupil dilation was reached on digits 1–9 and STM capacity, as reflected in our STM task and the WISC Digit Span subtest. That is, individual children or adults whose pupils peaked later in the encoded sequence were more likely to have a higher STM span, as reflected in multiple measures. This pupil-behavior relationship, observed independently of age group, is all the more noteworthy because performance on our STM overload task was not significantly related to WISC forward span after partialing out the effect of group. Thus, pupillometry reveals a relationship between encoding on one task and recall on another that would not have been detected via comparison of behavioral performance on the two tasks. These findings suggest that the allocation of cognitive resources—what Kahneman (1973) called the “intensive aspect” of attention—during encoding of information at high cognitive loads is an important contributor to the development of STM.

However, the group difference in STM performance suggests that attention is not the only factor. The groups exhibited the same rate of dilation for digits 1 through 6, indicating a similar level of cognitive effort on those digits, yet adults outperformed children on recall for all digits, not just digits 7 and higher. Thus, similar levels of cognitive resource allocation in children and adults could not fully account for the group difference in recall performance (also Karatekin, 2004). Success on the digit span task requires participants to maintain encoded digits in STM while additional digits are presented, as well as during the recall phase. Attention, echoic memory, rehearsal, and mnemonic strategies are all components of maintenance that contribute to STM performance, and it is likely that each of these cognitive components contributes to the more global measure offered by the task-evoked pupillary response. Further, STM capacity is operationalized in the digit span task as the number of digits that one can accurately recall in the right order via verbal report. This number is likely to be smaller than the number of digits in a sequence that one could accurately identify as “old” on a test of recognition memory (e.g., Unsworth and Engle, 2007b). Pupillometry has been employed in the context of long-term recognition memory (for review see Goldinger and Papesh, 2012), and given the relationship we have found between peak pupil dilation and STM span, it would be of interest to examine how the dynamics of pupil dilation and constriction at encoding relate to subsequent recognition memory as well as recall.

In summary, this study provides insight into the unique relationship between task engagement at encoding and STM capacity, and highlights the role that pupillometry can play in elucidating developmental changes and individual differences in cognition. This work supports Simmering and Perone's (2013) thesis that measures of “micro-behaviors” combined with “macro” performance measures can inform research on cognitive development. Our results further highlight the potential of pupillometry to address inquiries that extend well beyond the study of prototypical adult cognition.

The methodological approach reported here also has practical applications. Our STM overload task could provide insights regarding the cognitive deficits observed in specific patient populations (e.g., in amnesics, Laeng et al., 2007)—or, perhaps in the future, in individual patients. More generally, the task-evoked pupillary response could in theory be used to evaluate the effectiveness of a targeted cognitive intervention, pinpointing precisely at what stage(s) of a task the intervention influences cognitive processing.

## Author note

This study was supported by a James S. McDonnell Foundation Scholar Award and a Tourette Syndrome Association research grant to Silvia A. Bunge. The Bunge lab's first eyetracking study could not have been completed without the help of numerous individuals. We thank Jordan Tharp and Galen Mancino for assistance with programming and set-up of the eyetracker, Jesse Niebaum, Jordan Tharp, and Farida Valji for assistance collecting and compiling data, Se Ri (Sally) Bae for writing up preliminary results for an undergraduate honor's thesis, Dr. Robert DiMartino for helpful discussions, and Marcus Stoiber and Dr. Davide Risso for invaluable consultation regarding data preprocessing.

### Conflict of interest statement

The authors declare that the research was conducted in the absence of any commercial or financial relationships that could be construed as a potential conflict of interest.

## Footnotes

^1^Three adults and one child who reported having taken medications on the day of testing were excluded from the current sample. Two adults took an antihistamine and one took Flomax; the child's medication is not known. Six of the young adults recruited through the UC Berkeley Research Participant Pool did not provide their exact ages.

^2^A wider range of data points, up to 700 ms on pupil diameter and 500 ms on distance, was used on datasets with fewer recorded data points, as required by the loess model.
